# Golf Club Selection with AI-Based Game Planning

**DOI:** 10.3390/e26090800

**Published:** 2024-09-19

**Authors:** Mehdi Khazaeli, Leili Javadpour

**Affiliations:** 1 School of Engineering and Computer Science, University of the Pacific, Stockton, CA 95211, USA; 2 Eberhardt School of Business, University of the Pacific, Stockton, CA 95211, USA

**Keywords:** sport analytics, AI, golf club selection, game planning

## Abstract

In the dynamic realm of golf, where every swing can make the difference between victory and defeat, the strategic selection of golf clubs has become a crucial factor in determining the outcome of a game. Advancements in artificial intelligence have opened new avenues for enhancing the decision-making process, empowering golfers to achieve optimal performance on the course. In this paper, we introduce an AI-based game planning system that assists players in selecting the best club for a given scenario. The system considers factors such as distance, terrain, wind strength and direction, and quality of lie. A rule-based model provides the four best club options based on the player’s maximum shot data for each club. The player picks a club, shot, and target and a probabilistic classification model identifies whether the shot represents a birdie opportunity, par zone, bogey zone, or worse. The results of our model show that taking into account factors such as terrain and atmospheric features increases the likelihood of a better shot outcome.

## 1. Introduction

The increasing interest in sports analytics over the last two decades can be attributed to advances in technology, where data have been used by teams and individuals to gain a competitive advantage. Statistics have always played a role in sport, but the use of predictive analysis has increased in recent years. The volume of data collected for each game creates a big data problem as it is not currently feasible to gain meaningful insights from raw data. Data-driven decision making is being incorporated into different aspects of sports, for example, in gambling and fantasy leagues, to improve team dynamics and performance and decision making and prevent injuries, etc. Deep learning and machine learning techniques are critical techniques, as data are unstructured and lack context.

Rapid advancements in artificial intelligence and machine learning have revolutionized the world of sports analytics. While analytics have been widely applied to sports like football [1,2,3], soccer [4,5], and basketball [6,7,8], golf has remained relatively untouched. Golf, as a sport, generates a significant amount of data from each shot, including club selection, launch angle, ball speed, and trajectory. The integration of AI, machine learning, and data-driven approaches has undoubtedly opened up new frontiers in golf analytics, offering golfers and coaches tools to enhance player performance, optimize club selection, and develop more effective game-planning strategies.

Golf is a precision sport involving various physical and mental skills that allow a player to navigate a course and aim to complete it in the fewest number of strokes. By analyzing data from past rounds, players can make informed decisions about shot selection and strategy. Additionally, tracking weather conditions, terrain, and equipment performance through analytics can further optimize a player’s approach, leading to more consistent and successful outcomes.

In this research, we are focusing on a model that assists players in selecting the best club for a given scenario. The system considers factors such as distance, terrain, wind strength and direction, and quality of lie as well as the player’s maximum shot data for each club.

## 2. Literature Review

There has been growing interest in adopting advanced data science and artificial intelligence techniques to gain deeper insights into golf performance [9]. These data can be leveraged using sophisticated statistical and machine learning models to uncover patterns and make data-driven decisions [10,11]. One key area of focus has been the use of sensor-based technologies and wearable devices to capture real-time data on a golfer’s movements, club dynamics, and shot trajectories [12]. By leveraging these data and applying advanced analytics, researchers have been able to identify patterns, predict shot outcomes, and provide personalized recommendations to golfers. For example, AI-powered systems can analyze a golfer’s swing mechanics, club–ball interaction, and environmental factors to suggest the most appropriate club selection for a given shot, taking into account the golfer’s skill level, course conditions, and desired ball trajectory [12].

By studying the long-term dependencies and causal relationships between shots, researchers have developed models that can accurately quantify the impact of each shot on a golfer’s overall performance. This information can be invaluable for coaches, players, and analysts in understanding the critical factors that contribute to successful golf outcomes and can inform training programs, strategic decision making, and performance evaluation [12].

Sharp [13] explores the mechanics of a successful golf swing, focusing on maximizing club-head speed upon impact. Through a review of existing research and the use of a simulation model, the author highlights the importance of factors such as physical attributes and swing timing in achieving optimal driving distance [13]. Meanwhile, Keogh and Hume [14] emphasize the role of biomechanical analysis and motor learning principles in enhancing golf performance.

Hollaus et al. [15] have demonstrated the use of smart technologies to provide real-time feedback to golfers. They explored the use of motion sensor mounted on the shaft of a golf club to detect the impact location on the clubface during a swing. Zhou et al. [16] used 3D kinematic analysis to highlight the importance of rotational biomechanics in golf, focusing on how consistent pelvis and upper torso movements among professionals contribute to optimal swing performance. Betzler et al. [17] examined how various impact factors, such as clubhead speed and ball contact location, influence ball flight outcomes and overall golf performance and found strong correlations between these factors, particularly when using long irons and drivers, which significantly affect a player’s game.

Drappi and Ting Keh [18] explore the application of statistical modeling to predict golf scores by analyzing individual shots. The authors utilize a dataset of shot-level data to develop and evaluate their predictive model, aiming to provide insights into the factors influencing golf performance at a granular level. Another study by Verikas et al. [19] examines electromyographic patterns during golf swings, analyzing the activation sequence of muscles involved and exploring if these patterns can predict the effectiveness of a golf shot. Additionally, Couceiro et al. [20] present a computer vision methodology to analyze the variability in golf putting techniques. By tracking putter movement and applying estimation algorithms, the study identifies unique putting “signatures” for individual players. Kim and Park [21] propose two machine learning methods for segmenting a golf swing into five phases using data from a single inertial measurement unit worn on the body. The authors tested their methods on data from professional and skilled male golfers and found that both methods were able to accurately segment the golf swings with minimal error.

These studies demonstrate the potential of leveraging data science, biomechanics, and AI-based techniques to gain a deeper understanding of the factors that contribute to successful golf performance. By integrating these approaches, researchers and practitioners can develop more effective training programs, club selection strategies, and game-planning tools to help golfers of all skill levels improve their performance on the course.

## 3. Materials and Methods

This study aims to assist golfers in decision making regarding club selection for the approach shot—a shot played either from the fairway or the rough prior to reaching the green. The methodology is divided into three sections:

- *Player Rank Calculation*: Each player was assigned a rank used to predict their shot performance.
- *Data*: Data from 745 individual shots collected by NCAA Division I men golfers were used. Monte Carlo simulation was then employed to generate 755 additional data, bringing the total data used to 1500.
- *Shot Prediction*: Given a specific shot scenario, the player’s chosen club determined the predicted outcome of the shot.

### 3.1. Player Rank Calculation

Each player was assigned a rank used for predicting their shot results. The rank for each player was calculated based on their input information. The feature vector collected from each player consisted of five attributes, which were used to calculate the player’s rank (Table 1).

Each time the player updated their data, a new rank was created. To calculate a new rank, we weighed each player’s normalized values and regressed them to the mean of the prior value. Drappi and Ting Keh [18] proposed a method for identifying the weights for golfers’ features, which we incorporated here to calculate a rank for each player.

(1) $$Rank=\frac{\left(Prior\;Rank+\sum \omega_{t}*\;f_{t}\right)}{Prior\;Weight\;of\;Rank+\sum \omega_{t})},$$

where $\omega_{t}$ is the weight used in the work of Drappi and Ting Keh [18]. $f_{t}$ is the player feature value from Table 1.

### 3.2. Data Collection

The data for this pilot study were collected from various male golfers playing in NCAA Division I, encompassing a total of 745 individual shots.

The feature vector included a set of 27 attributes, which are detailed in Table 2 and can be categorized as follows:

- *Shot Scenario (20 attributes):* these attributes relate to pin location, lie, and adjustments.
- *Shot Selection (5 attributes):* these include attributes such as club, shape, curve intention, target, and swing.
- *Shot Result (2 attributes):* these identify the quality of the shot and its relative location to the hole (e.g., birdie opportunity, par zone, bogey zone, etc.).

**Figure 1:**
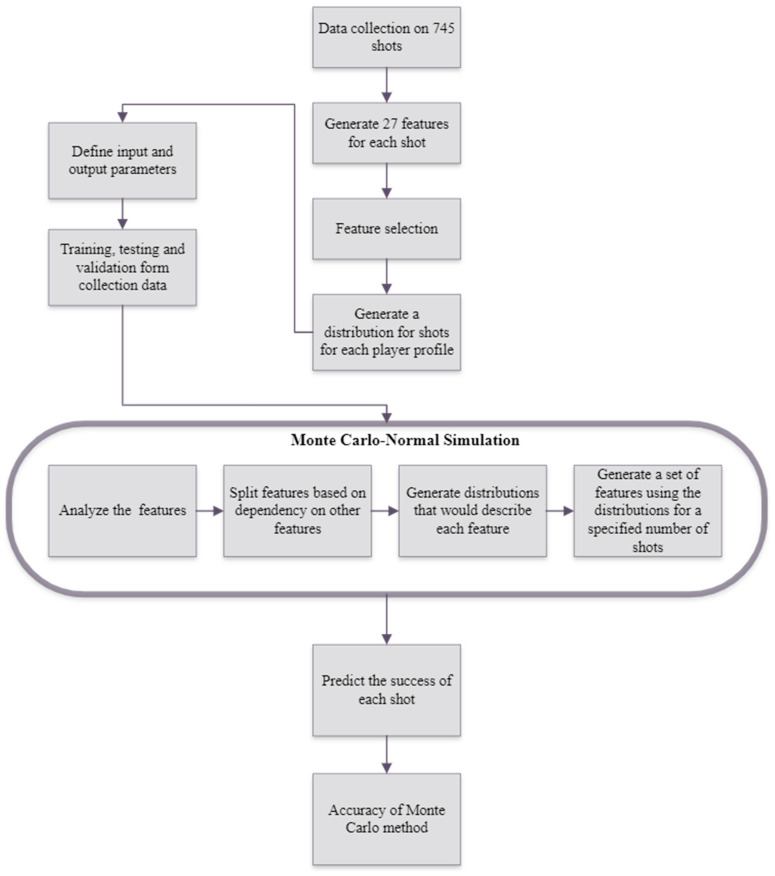
Monte Carlo process.

Monte Carlo simulation was used to generate additional data, resulting in a total of 1500. The diagram below illustrates this process (Figure 1).

The combination of data collection, feature generation, attribute correlation, and Monte Carlo–normal simulation allowed for a robust approach to enhancing the decision-making process of golf club selection.

### 3.3. Modeling and Shot Prediction

In this proposed AI-based game, the player provides their golfer skills data, which are then used to calculate a scoring rank (as detailed in Section 3.1). This rank ensures that correct training data are used to predict the outcome of the shot.

In golf, understanding the distance one of the clubs can achieve helps in calculating the distances achievable by all other clubs. There is a well-established relationship between the distances each club can achieve. This relationship allows players to make informed decisions about club selection during play, optimizing their performance by accurately predicting the distance each shot will travel with different clubs.

It is worth noting that as players select their preferences for clubs to the finest detail, they can have a maximum of 14 clubs in their bag at a time. We excluded the putter and driver as we only considered approach shots. Based on data collected from NCAA players, we identified the following 14 clubs, as listed in Table 3, in our dataset.

To provide more insight for the player during the game, the average yardage for each of the 14 clubs was calculated based on their 7-iron yardage. The commonly recognized golf club names associated with each yardage are shown in Table 3, along with the formula used to calculate the average yardage. These data were generated based on NCAA averages for men’s teams. By incorporating these calculations, the base distance was established for the model to carry out the optimal club selection.

During the game, an approach shot scenario was given. Based on the distance to the hole and the data in Table 3, the game identified the four best club choices. The player then chose their club of choice, shot, and target for the scenario provided. This input was then used in our model to predict the outcome of the shot.

In this paper, we hypothesize that while knowing the yardage distance of your 7-iron can help calculate distances for other clubs, incorporating other variables such as terrain and atmospheric conditions will lead to a more accurate prediction of shot outcomes. Our framework aims to build a predictive model that accounts for these factors, ultimately providing better guidance for club selection and improving overall performance in the approach shot. By integrating these additional elements, our model strives to enhance the precision and reliability of predictions, enabling players to make more informed decisions and optimize their game strategy.

## 4. Results

We trained our model on a dataset of 1500 approach shots to demonstrate the importance of atmospheric and terrain features. We compared the out-of-sample (OOS) cross-entropy errors for 300 test data for four models under different scenarios: (1) only the distance to the hole feature, (2) distance and atmospheric features, (3) distance and terrain features, and (4) all features. By evaluating these different scenarios, we aimed to illustrate how incorporating additional relevant features can improve model performance. Examining the results, we clearly saw that by incorporating more features in our model, the entropy error decreased (Table 4).

After generating the test feature vector for each shot, Python 3.12.6, Altair RapidMiner (https://altair.com/altair-rapidminer), and JMP Pro (predictive analytics software, https://www.jmp.com/en_us/software/predictive-analytics-software.html) were used for data analysis. Predictive modeling was used to predict the outcome of the shot as a birdie opportunity, par zone, bogey zone, or double plus. Using a prediction model, we built a machine learning model to predict the outcome of the shot based on all the features listed in Table 2. We evaluated the accuracy for three different pattern recognition models and chose a multi-level neural network for learning non-linear relationships (Table 5). As the deep learning algorithm is computationally expensive, we opted for a 80:20 split between training and test data. The multilayer feed-forward artificial neural network model was built using an H_2_O deep learning algorithm and was set up with a rectifier activation function and ten epochs. The model predicted the actual result of the shots with an accuracy of 72%.

With the use of these predictions, players can learn how to optimize each shot by choosing the best club according to atmospheric and terrain features. Information gain was used to calculate the weight of each of the attributes in predicting the outcome of the shot. The normalized weights by information gain are shown in Table 6, highlighting the importance of various factors in determining the outcome of the shot. This analysis demonstrated that incorporating a range of variables beyond just distance could significantly enhance the accuracy of shot outcome predictions, ultimately leading to better performance and strategy on the golf course.

## 5. Conclusions and Future Work

Our model was designed to predict the outcome of an approach shot given a specific scenario. The player input their chosen club, shot, and target and the model predicted whether the outcome of the shot was a birdie opportunity, par zone, bogie zone, or double plus. This is specifically important for players who practice their club selection by taking into consideration not only the distance to the hole but other atmospheric and terrain features. Table 6 provides the information gain for the features used and shows that features other than distance are important in selecting the correct club and achieving a good outcome.

In future work, we will collect data from players from different levels and test our model in order to identify whether the same result stands (or not) for players of different skill levels.

## Figures and Tables

**Table 1:** Player features used to calculate player’s rank.

Player Feature	Description
Scoring average	Adjusted scoring average
7-iron distance	Average driving distance (yards)
7-iron approximate dispersion width	Mean deviation of shots from flag (lateral dispersion) in yards
7-iron approximate dispersion depth	Mean deviation of shots from flag (longitudinal dispersion) in yards
SG: short game	Strokes gained for short approach shot

**Table 2:** Feature vector collected for each individual shot.

Category	Attribute
Pin location	Distance to hole
	Distance from right side
	Distance from left side
	Distance from front edge
	Distance from back edge
	Width between penalty areas
	Width of playing position
Lie	Fairway/rough/bunker
	Fairway type
	Rough length
	Quality of lie
	Uphill/downhill/flat
	Side hill
Adjustments	Elevation
	Uphill/downhill2
	Temperature
	Wind direction
	Wind strength
Obstacles	Recovery situation
	Severity of recovery
	Forced Cary
Shot selection	Club
	Shape (straight, draw, cut)
	Curve intention
	Target (left, straight, right)
	Swing (full, 3/4, half shot)
Shot result	Quality of shot
	Distance long/short of target (birdie opportunity, par zone, bogey zone, double plus)

**Table 3:** Distance calculation for each club based on the 7-iron average yardage.

Player Feature	Description	Distance (yds)
*j = 1*	3-wood	${Dist}_{j=7}+73$
*j = 2*	3-hybrid	${Dist}_{j=7}+55$
*j = 3*	3-iron	${Dist}_{j=7}+42$
*j = 4*	4-hybrid	${Dist}_{j=7}+36$
*j = 5*	4-iron	${Dist}_{j=7}+33$
*j = 6*	5-iron	${Dist}_{j=7}+23$
*j = 7*	6-iron	${Dist}_{j=7}+12$
*j = 8*	7-iron	${Dist}_{j=7}$
*j = 9*	8-iron	${Dist}_{j=7}-12$
*j = 10*	9-iron	${Dist}_{j=7}-24$
*j = 11*	pitching wedge	${Dist}_{j=7}-34$
*j = 12*	gap wedge	${Dist}_{j=7}-47$
*j = 13*	sand wedge	${Dist}_{j=7}-60$
*j = 14*	lob wedge	${Dist}_{j=7}-70$

**Table 4:** Model results.

Model	OOS Cross Entropy Error
(Benchmark) Distance feature only	1.097
Distance + atmospheric features	1.066
Distance + terrain features	0.965
All features	0.843

**Table 5:** Different models’ accuracy.

Model	Accuracy	Standard Deviation	Total Time
Deep Learning	72%	±1.6%	5 s
Gradient Boosted Trees	67%	±3.2%	29 s
Naïve Bayes	65%	±6%	3 s
Random Forest	69%	±2.3%	10 s
eXtreme Gradient Boosting	70	±4%	20 s

**Table 6:** Attribute weights by information gain.

Attribute	Normalized Weight
Distance to hole	1
Width of playing position	0.772
Club	0.672
Quality of lie	0.578
Distance from left side	0.515
Fairway/rough/bunker	0.396
Uphill/downhill2	0.308
Wind strength	0.222
Width between penalty areas	0.206
Side hill	0.167
Recovery situation	0.151
Distance from back edge	0.129
Uphill/downhill/flat	0.121
Shape	0.110
Curve intention	0.093
Distance from right side	0.085
Wind direction	0.083
Temperature	0.078
Distance from front edge	0.077
Fairway type	0.043
Rought length	0.024
Target (Left/ Right/ Flag)	0.004

## Data Availability

Data will be provided upon request.

## References

[1] Anderson C., Sally D. (2020). The role of analytics in football: The rise of data-driven approaches. J. Sports Anal..

[2] McHale I., Scarf P. (2011). Predictive modelling in professional football: Evidence from the English Premier League. J. Oper. Res. Soc..

[3] Tuyls K., Omidshafiei S., Müller P., Wang Z., Connor J.J., Hennes D., Graham I., Spearman W., Waskett T., Steel D.I. (2021). Game Plan: What AI can do for Football, and What Football can do for AI. AI Access Found..

[4] Fernández J., Bornn L. (2018). Spatial analysis of player positioning in professional soccer. J. Quant. Anal. Sports.

[5] Caley M., Lawrence T. (2017). Expected Goals (xG) and soccer analytics: A review of applications and methodologies. J. Sports Anal..

[6] Sarlis V., Tjortjis C. (2020). Sports analytics—Evaluation of basketball players and team performance. Inf. Syst..

[7] Javadpour L., Blakeslee J., Khazaeli M., Schroeder P. (2022). Optimizing the best play in basketball using deep learning. J. Sports Anal..

[8] Terner Z., Franks A. (2021). Modeling player and team performance in basketball. Ann. Rev. Stat. Appl..

[9] Hurley W.J. (2011). Operation Research in Golf.

[10] Alegre I., Canela M.Á., Pastoriza D. (2022). Dataset on PGA Tour tournament entry. Data Brief.

[11] Khan M.A., Habib M., Saqib S., Alyas T., Masood K., Ghamdi M.A.A., Almotiri S.H. (2020). Analysis of the Smart Player’s Impact on the Success of a Team Empowered with Machine Learning. Comput. Mater. Contin..

[12] Chidambaram S., Maheswaran Y., Patel K., Sounderajah V., Hashimoto D.A., Seastedt K.P., McGregor A.H., Markar S.R., Darzi A. (2022). Using Artificial Intelligence-Enhanced Sensing and Wearable Technology in Sports Medicine and Performance Optimisation. Sensors.

[13] Sharp R.S. (2008). On the mechanics of the golf swing. Proc. R. Soc. A Math. Phys. Eng. Sci..

[14] Keogh J., Hume P. (2012). Evidence for biomechanics and motor learning research improving golf performance. Sports Biomech..

[15] Hollaus B., Heyer Y., Steiner J., Strutzenberger G. (2023). Location matters—Can a smart golf club detect where the club face hits the ball?. Sensors.

[16] Zhou J.Y., Richards A., Schadl K., Ladd A., Rose J. (2022). The swing performance index: Developing a single-score index of golf swing rotational biomechanics quantified with 3D kinematics. Front. Sports Act. Living.

[17] Betzler N.F., Monk S.A., Wallace E.S., Otto S.R. (2012). An assessment of the relationships between ball flight results, impact factors, and golf performance. J. Sports Sci..

[18] Drappi C., Ting Keh L.C. (2019). Predicting golf scores at the shot level. J. Sports Anal..

[19] Verikas A., Vaičiukynas E., Gelžinis A., Parker J., Olsson M.C. (2016). Electromyographic patterns during golf swing: Activation sequence profiling and prediction of shot effectiveness. Sensors.

[20] Couceiro M.S., Portugal D., Gonçalves N., Rocha R.P., Luz JM A., Figueiredo C.M.S., Dias G. (2012). A methodology for detection and estimation in the analysis of golf putting. Int. J. Comput. Vis..

[21] Kim M., Park S. (2020). Golf Swing Segmentation from a Single IMU Using Machine Learning. Sensors.

